# Traditional Chinese Medicine Compounds Containing *Lonicera japonica*, *Chrysanthemum morifolium*, and *Siraitia grosvenorii* Inhibits the Growth of *Streptococcus mutans*

**DOI:** 10.1155/2022/5802343

**Published:** 2022-10-13

**Authors:** Xiaomin Zhou, Asimuguli Reheman, Zhongjie Kang, An Long, Tingting Wang

**Affiliations:** ^1^Department of Anaesthesia, Obstetrics and Gynecology Hospital of Fudan University, Shanghai, China; ^2^Department of Anaesthesia, Kashgar Regional Second People's Hospital, Xinjiang Uygur Autonomous Region, Kashgar, China; ^3^Department of Anaesthesia, Changning Maternity and Infant Health Hospital, Shanghai, China

## Abstract

Postoperative sore throat (POST) is one of the common complications after endotracheal intubation under general anesthesia. This work attempted to design and screen out the traditional Chinese medicine (TCM) compounds containing *Lonicera japonica* Thunb., *Chrysanthemum morifolium* Ramat., and *Siraitia grosvenorii* (Swingle) C. Jeffrey ex A. M. Lu et Z. Y. Zhang, and further explored its antibacterial effect on POST. The antibacterial activities of *Lonicera japonica*, *Chrysanthemum morifolium*, and *Siraitia grosvenorii* on *Streptococcus mutans* (*S*. *mutans*) UA159 was measured. *Lonicera japonica* (40 mg/mL), *Chrysanthemum morifolium* (10 mg/mL), and *Siraitia grosvenorii* (10 mg/mL) effectively inhibited the growth of *S*. *mutans*. Then, 5 different TCM compounds were designed using the U5 (5^3^) uniform design experimentation method. TCM compound C3 (50 mg/mL *Lonicera japonica*, 10 mg/mL *Chrysanthemum morifolium*, and 15 mg/mL *Siraitia grosvenorii*) significantly promoted the inhibition zone and inhibited the biofilm formation of *S. mutans*. In addition, TCM compound C3 exerted great antibacterial effects on *S. mutans* and had the least effect on cell viability of primary human pharyngeal epithelial cells. In conclusion, this work demonstrated that TCM compound containing *Lonicera japonica*, *Chrysanthemum morifolium*, and *Siraitia grosvenorii* inhibited the growth of *S*. *mutans*. Thus, this study provides a theoretical basis for screening out the optimal compatibility of effective components in TCM mouthwash for relieving POST.

## 1. Introduction

Patients with general anesthesia require endotracheal intubation to facilitate airway management during anesthesia and prevent foreign bodies from entering the airway [1]. Endotracheal intubation during general anesthesia, postoperative extubation, and poor physical activity during surgery damage patients' airway mucosa and cause postoperative sore throat (POST) [2]. The main clinical manifestations of POST are redness, swelling and congestion in the throat, varying degrees of pain, hoarseness, and foreign body sensation in the throat [3]. The occurrence of these conditions all affects the satisfaction of patients after surgery, and even influences the patients' diet and causes fatal injuries. The previous study has shown that up to 62% of patients with general anesthesia experience varying degrees of POST after endotracheal intubation [4]. Supraglottic airway devices are widely used in general anesthesia because of its advantages of small damage and convenient use. Nevertheless, regardless of the type of supraglottic airway devices, the incidence of POST in patients remains as high as 50% [5]. Therefore, POST has been paid more and more attention by both doctors and patients.

The causes of POST are closely related to abnormal bacterial reproduction. Due to the influence of surgical pressure and anesthetic drugs, the immune function of patients during the operation is limited. At this time, oropharyngeal bacteria reproduce abnormally, causing damage to the oral mucosa, and may even lead to ulcers, acute pharyngitis, and ultimately POST [6]. Among them, *Streptococcus mutans* (*S*. *mutans*) is the highest proportion of natural flora in the oral cavity and is essential in initiating factors for the occurrence and development of POST [7]. Therefore, *S*. *mutans* is the key to treat POST. The most commonly used method is drug treatment, such as inhaled fluticasone propionate, intravenous dexamethasone, and taking streptococcus tablets [8–10]. Each method has its limitations and uncertain success rates and is therefore not routinely used in clinical practice. Thus, there is an urgent need for a clear and simple method to reduce POST.

The previous study has confirmed that traditional Chinese medicine (TCM) has effective remedy on sore throat [11]. *Lonicera japonica* Thunb., *Chrysanthemum morifolium* Ramat., and *Siraitia grosvenorii* (Swingle) C. Jeffrey ex A. M. Lu et Z. Y. Zhang. are common TCMs with strong biological activity, which have anti-inflammatory and immune-regulating effects. TCM preparations containing these drugs have good effects in the treatment of pharyngitis [12, 13]. Our previous study has confirmed that chewing gum containing *Lonicera japonica*, *Chrysanthemum morifolium*, and *Siraitia grosvenorii* before surgery significantly reduces the incidence of POST [14]. In addition, mouthwash is another way to cleanse mouth and keep bacteria in oropharynx from multiplying. Mouthwashes and chewing gums containing the same ingredients inhibit the proliferation and biofilm formation of *S*. *mutans* to a similar extent [15]. Therefore, this work designed and screened out the optimal compatibility of TCM containing *Lonicera japonica*, *Chrysanthemum morifolium*, and *Siraitia grosvenorii* and further explored its antibacterial effects on POST.

## 2. Materials and Methods

### 2.1. Preparation of Drug

Volatile oil extraction method was used to extract the effective ingredients of each TCM. Flowers of *Lonicera japonica* (50 g), flowers of *Chrysanthemum morifolium* (50 g), and fruit of *Siraitia grosvenorii* (50 g) were soaked in 1000 mL of distilled water for 20 min. Then, the soaked solution was condensed and refluxed for 6 h, and the liquid medicine and volatile oil were collected. The liquid medicine was immediately centrifuged at 3600 rpm for 15 min, and then the liquid supernatant was collected. The supernatant was mixed with the volatile oil, 0.2 g sodium citrate, and 0.5 mL Tween-80 to mix well and dissolve. The solution was concentrated to 100 mL, and then *Lonicera japonica*, *Chrysanthemum morifolium*, and *Siraitia grosvenorii* extracting solutions with a concentration of 500 mg/mL was obtained. The TCM solution was stored at 4°C for further use. Herbarium numbers of *Lonicera japonica*, *Chrysanthemum morifolium*, and *Siraitia grosvenorii* are 20140830265 (National Specimen Information Infrastructure, NSII), 5334 (NSII), and 5307210549 (NSII), respectively. The plant identification details of *Lonicera japonica*, *Chrysanthemum morifolium*, and *Siraitia grosvenorii*were shown in Supplementary materials.

### 2.2. Bacterial Strains and Growth Conditions

*S. mutans* UA159 was cultured in brain heart infusion (BHI; Solarbio, Beijing, China) broth or BHI agar plate containing sheep blood with 1 g/L spectinomycin (Solarbio) in an anaerobic incubator (Thermo Fisher Scientific, Waltham, MA, USA). The anaerobic conditions were 90% N_2_, 5% CO_2_, 5% H_2_, and 37°C.

### 2.3. Antibacterial Activity Analysis

*Lonicera japonica*, *Chrysanthemum morifolium*, and *Siraitia grosvenorii* extracting solutions, 0.2 g sodium citrate combined with 0.5 mL Tween-80 were dissolved in the BHI-sheep blood agar plate separately, and the final drug concentration was 10, 20, 30, 40, and 50 mg/mL. *S. mutans* were cultured in BHI broth for 24 h. The concentration of *S. mutans* was detected utilizing hemocytometer, and then adjusted to the appropriate concentration (1 × 10^5^ cells/mL or 2 × 10^6^ cells/mL). *S. mutans* (100 *μ*L) was seeded into the drug-contained BHI-sheep blood agar plate and incubated under anaerobic conditions at 37°C for 48 h. The number of colonies of *S. mutans* (CFU/mL) was calculated.

### 2.4. Uniform Design Experimentation

*Lonicera japonica* (X), *Chrysanthemum morifolium* (Y), and *Siraitia grosvenorii* (*Z*) extracting solutions were considered as 3 factors, and each drug extract was divided into 5 concentrations. According to U5 (5^3^) uniform design experimentation method, the experiment was uniformly designed and fitted to 5 different TCM compounds (Table 1).

### 2.5. Inhibition Zone Assay

*S. mutans* at a concentration of 2 × 10^6^ cells/mL was evenly spread on the BHI-sheep blood agar plate, and let it stand for 5 min until the bacterial liquid was completely absorbed. Sterile filter papers containing different concentrations of TCM compounds (C1, C2, C3, C4, and C5) were placed on the plates, and the filter papers containing sterilized water were served as control. The plates were incubated under anaerobic conditions at 37°C for 48 h. After that, the diameter of the inhibition zone of each group was measured using a vernier caliper.

### 2.6. Crystal Violet Staining

*S. mutans* was seeded into a 96-well plate containing BHI-sheep blood broth and different concentrations of TCM compounds (C1, C2, C3, C4, and C5). *S. mutans* was incubated under anaerobic conditions at 37°C for 24 h. After that, the bacteria suspension in each well was discarded, and then the plate was washed with distilled water for 3 times. Each well was stained with 1 g/L crystal violet for 15 min, and then incubated with ethanol: acetone (4 : 1) mixture. Finally, the absorbance of each well at 570 nm was measured on a Multiskan microplate reader (Thermo Fisher Scientific, Waltham, MA, USA). The images of biofilm were taken under an optical microscope (Nikon, Tokyo, Japan) at 1000 × magnification.

### 2.7. Participants

This work recruited 10 female patients who undergoing hysteroscopic surgery at the Obstetrics and Gynecology Hospital of Fudan University. These patients (age > 18 years old) did not suffer from mucosal diseases or systemic diseases. All participants were informed and given written consent. The study protocols were carried out according to the Declaration of Helsinki and approved by the Ethics Committee of Obstetrics and Gynecology Hospital of Fudan University (number: 2022-82).

### 2.8. Cell Culture

Primary human pharyngeal epithelial cells were separated from the throat swabs of the patients. The throat swab was collected from patients under aseptic conditions, and then incubated in Dulbecco's modified eagle medium (DMEM; Thermo Fisher Scientific, Waltham, MA, USA) containing 1 mg/mL dispase (Roche, Basel, Switzerland) at 37°C and 5% CO_2_. After 1 h of digestion, the digested cells were washed with DMEM for several times. Cells were then cultured in DMEM supplemented with 10% fetal bovine serum (FBS; Thermo Fisher Scientific) at 37°C and 5% CO_2_. The 3rd generation cells in good growth condition were used for further analysis.

### 2.9. CCK-8 Assay

For cell toxicity assay, primary human pharyngeal epithelial cells (5000 cells/100 *μ*L) were seeded into 96-well plates and cultured at 37°C and 5% CO_2_ for 24 h. Cells at the logarithmic phase were incubated with different concentrations of TCM compounds (C1, C2, C3, C4, and C5) at 37°C for 0, 12, 24, 48, or 72 h. Subsequently, cells were then incubated with CCK-8 reagent at 37°C for 1 h. For cell viability assay, cells at the logarithmic phase were cocultured with *S. mutans* at 37°C for 2 h, and then incubated with TCM compound C3 at 37°C for 12 h. Then, cells were incubated with CCK-8 reagent at 37°C for 1 h. Finally, the absorbance of each well at 450 nm was measured on a Multiskan microplate reader.

### 2.10. Coculture of Pharyngeal Epithelial Cells and *S. mutans*

Primary human pharyngeal epithelial cells were seeded into 96-well plates and cultured in DMEM for 24 h. Then, *S. mutans* was added into each well, and cocultured with pharyngeal epithelial cells at 37°C for 2 h. The coculture system of pharyngeal epithelial cells and *S. mutans* were seeded into the TCM compound C3-contained BHI-sheep blood agar plate and incubated under anaerobic conditions at 37°C for 24 h. The number of colonies of *S. mutans* (CFU/mL) was calculated.

### 2.11. Statistical Analysis

All protocols were performed for 3 times. Data were expressed as mean ± standard deviation and analyzed using SPSS 22.0 statistical software (IBM, Armonk, NY, USA). The two-tailed Student's *t*-test and one-way ANOVA were utilized to analyze the statistical difference. *P* value less than 0.05 was considered as a significant difference.

## 3. Results

### 3.1. The Antibacterial Effects of *Lonicera japonica*, *Chrysanthemum morifolium*, and *Siraitia grosvenorii* on *S. mutans*

**Figure 1:**
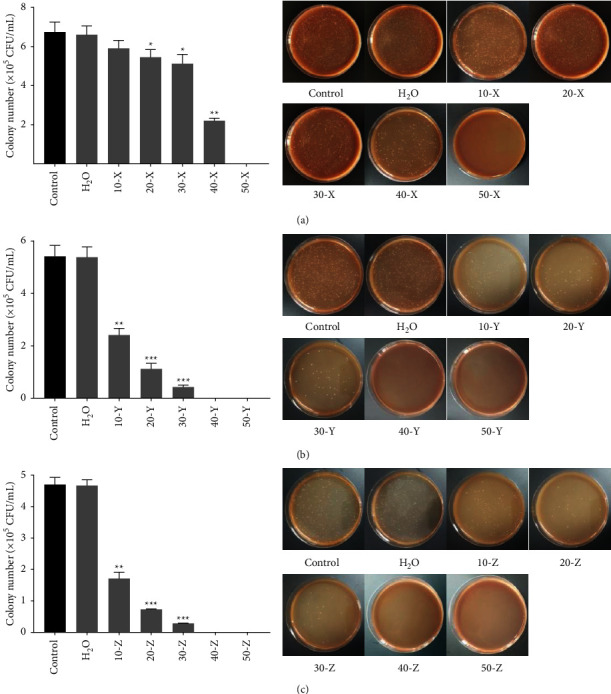
Antibacterial activity of *Lonicera japonica*, *Chrysanthemum morifolium*, and *Siraitia grosvenorii* on *S. mutans*. The antibacterial activity of different concentrations (10–50 mg/mL) of *Lonicera japonica* (a), *Chrysanthemum morifolium* (b), and *Siraitia grosvenorii* (c) on *S. mutans* was measured.  ^*∗*^ ^*∗*^*P* < 0.01 vs. control group.

We first determined the antibacterial effects of *Lonicera japonica*, *Chrysanthemum morifolium*, and *Siraitia grosvenorii* extracting solutions on *S. mutans*. The results of the antibacterial activity analysis revealed that 20 and 30 mg/mL of *Lonicera japonica* inhibited the growth of *S. mutans* slightly. *Lonicera japonica* at 40 mg/mL significantly inhibited the colony number of *S. mutans*, while *Lonicera japonica* at 50 mg/mL completely inhibited the growth of *S. mutans* (Figure 1(a)). *Chrysanthemum morifolium* repressed the colony number of *S. mutans* in a concentration-dependent manner. When *S. mutans* was treated with 40 and 50 mg/mL of *Chrysanthemum morifolium*, *S. mutans* was completely unable to grow (Figure 1(b)). *Siraitia grosvenorii* at 10, 20, and 30 mg/mL notably suppressed the growth of *S. mutans*. The growth of bacteria was completely inhibited by 40 and 50 mg/mL of *Siraitia grosvenorii* (Figure 1(c)). Sodium citrate and Tween-80 had no influence on the growth of *S. mutans* (Supplementary Figure 1).

### 3.2. The Antibacterial Effects of TCM Compounds on *S. mutans*

**Figure 2:**
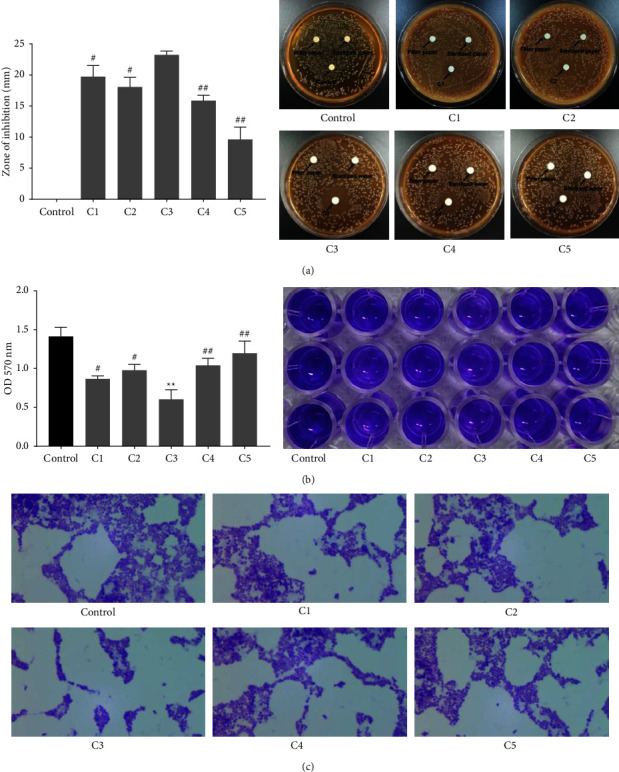
The antibacterial activity of TCM compound on *S. mutans*. (a) The inhibition zone assay detected the antibacterial activity of different concentrations of the TCM compounds (C1, C2, C3, C4, and C5) against *S. mutans*. (b) Crystal violet staining assessed the effect of different concentrations of the TCM compounds (C1, C2, C3, C4, and C5) on the biofilm formation of *S. mutans*. (c) Microscopic images of biofilm. 1000 × magnification.  ^*∗*^ ^*∗*^*P* < 0.01 vs. control group; ^#^*P* < 0.05, ^##^*P* < 0.01 vs. C3 group.

Then, we measured the antibacterial effects of 5 different TCM compounds on *S. mutans* by inhibition zone assay. As shown in Figure 2(a), different size of inhibition zone was formed around the 5 different TCM compounds on the plates. The zone of inhibition of C3 was larger than C1, C2, C4, and C5. Crystal violet staining was performed to assess the effect of 5 different TCM compounds on the biofilm formation of *S. mutans*. Compared with the control group, C3 significantly inhibited the biofilm formation of *S. mutans*. Compared with the C3 group, C1, C2, C4, and C5 promoted the biofilm formation of *S. mutans* (Figure 2(b)). Microscopic images of biofilm uncovered that 5 TCM compounds all inhibited the biofilm formation of *S. mutans*, especially TCM C3 (Figure 2(c)). Thus, TCM C3 exerted the best antibacterial effects on *S. mutans*.

### 3.3. The Antibacterial Activity of C3 on Coculture of Primary Human Pharyngeal Epithelial Cells and *S. mutans*

**Figure 3:**
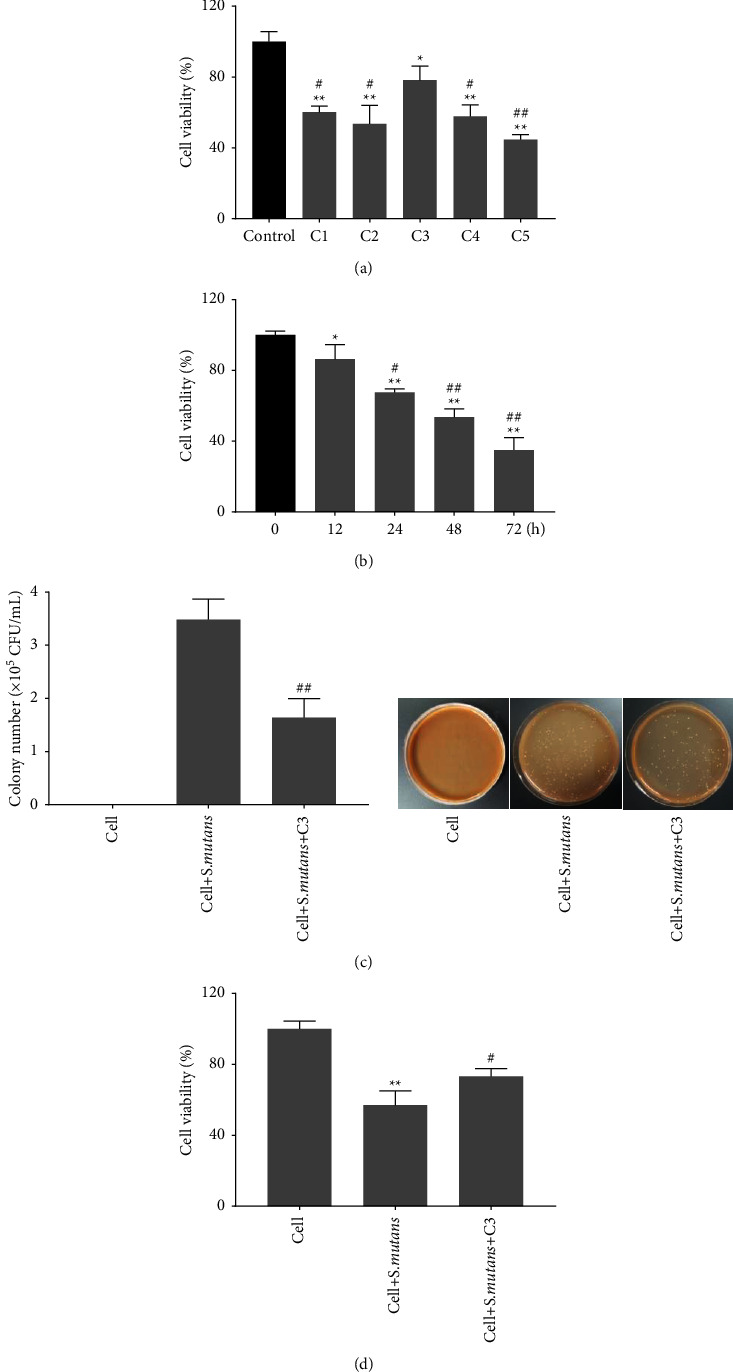
The antibacterial activity of C3 on coculture of primary human pharyngeal epithelial cells and *S. mutans*. (a–b) CCK-8 assay examined cell viability of primary human pharyngeal epithelial cells following the treatment of different concentrations of TCM compound (C1, C2, C3, C4, and C5) for 0, 12, 24, 48, or 72 h  ^*∗*^*P* < 0.05,  ^*∗*^ ^*∗*^*P* < 0.01 vs. control group; ^#^*P* < 0.05, ##*P* < 0.01 vs. C3 group. (c) The antibacterial activity of C3 on *S. mutans* in coculture of primary human pharyngeal epithelial cells and *S. mutans*. ^##^*P* < 0.01 vs. cell + *S. mutans* group. (d) CCK-8 assay detected cell viability of primary human pharyngeal epithelial cells in coculture of primary human pharyngeal epithelial cells and *S. mutans* following C3 treatment.  ^*∗*^ ^*∗*^*P* < 0.01 vs. cell group; ^#^*P* < 0.05 vs. cell + *S. mutan*s group.

The toxicity of TCM compounds on primary human pharyngeal epithelial cells was examined by CCK-8 assay. The results uncovered that TCM compound C1, C2, C3, C4, and C5 inhibited cell viability of primary human pharyngeal epithelial cells. Compared with TCM compound C1, C2, C4, and C5, the inhibitory effect of C3 on viability of primary human pharyngeal epithelial cells was milder (Figure 3(a)). TCM compound C3 exhibited inhibitory effect on cell viability of primary human pharyngeal epithelial cells after 12, 24, 48, and 72 h of treatment (Figure 3(b)). Thus, C3 was used to treat coculture of primary human pharyngeal epithelial cells and *S. mutans* for 12 h and measured the antibacterial activity of C3 on *S. mutans*. The colony number of *S. mutans* in coculture of primary human pharyngeal epithelial cells and *S. mutans* was obviously decreased (Figure 3(c)). The results obtained from CCK-8 assay showed that cell viability of primary human pharyngeal epithelial cells was significantly inhibited by *S. mutans*. C3 treatment impaired *S. mutans*-mediated inhibition of cell viability on primary human pharyngeal epithelial cells (Figure 3(d)). Therefore, TCM compound C3 exerted great antibacterial effects on *S. mutans* and had the least effects on cell viability of primary human pharyngeal epithelial cells.

## 4. Discussion

POST is one of the common complications after endotracheal intubation and extubation under general anesthesia. *Lonicera japonica*, *Chrysanthemum morifolium*, and *Siraitia grosvenorii* extracting solutions significantly inhibited growth of *S*. *mutans* at 40, 10, and 10 mg/mL, respectively. TCM compound C3 formed a large inhibition zone on the plate of *S. mutans* and repressed the biofilm formation of *S. mutans*. Moreover, TCM compound C3 exerted great antibacterial effects on *S. mutans* and had the least effects on cell viability of primary human pharyngeal epithelial cells. Thus, TCM compounds containing *Lonicera japonica*, *Chrysanthemum morifolium*, and *Siraitia grosvenorii* inhibited growth of *S*. *mutans*, which may alleviate POST.

Modern medical pharmacological experiments show that *Lonicera japonica* has broad-spectrum antibacterial, antiviral, anti-inflammatory, antipyretic, and immune-enhancing effects [16]. Minami et al. have found that *Lonicera japonica* flower bud extract enhances the phagocytic activity of macrophages, increases the survival rate, and reduces *Citrobacter rodentium* colonization in *Citrobacter rodentium*-induced digestive tract infection mouse model [17]. The polysaccharide extracted from *Lonicera japonica* exerts neuroprotective effect to improve behavioral changes and inhibit neuronal loss in lipopolysaccharide-induced learning and memory impairment mice [18]. *Lonicera japonica* represses activation of hepatic stellate cells, reduces epithelial-mesenchymal transition, and liver oxidative stress injury through regulating Nrf2 pathway, which contributes to attenuate CCl_4_-induced liver fibrosis in mice [19]. In this work, we first confirmed the antibacterial activities of *Lonicera japonica* against *S*. *mutans*.

*Chrysanthemum morifolium* is a TCM raw material for clinical medicine and tea beverages, and its pharmacological effects are cardiovascular protection, antitumor, blood lipid regulation, liver protection, and labor pain [20]. *Chrysanthemum morifolium* water extract inhibits differentiation of bone marrow-derived macrophages by regulating PLC*γ*2/CREB/c-fos/NFATc1 signaling pathway, thereby alleviating osteoporosis [21]. *Chrysanthemum morifolium* exhibits a notable antitrypanosomal activity and antimicrobial activity versus *Streptococcus agalactiae* and inhibits replication of vesicular stomatitis virus, which may be served as an antiinfective agent in different food products and pharmaceutical preparations [22]. This work revealed the antibacterial activities of *Chrysanthemum morifolium* against *S*. *mutans*.

*Siraitia grosvenorii* contains a large amount of flavonoids, phenolic acids, and terpenoids as well as carbohydrate, protein, amino acid, and vitamin components [13]. *Siraitia grosvenorii* and its extracts have reactive oxygen species scavenging, anti-inflammatory, antifatigue, and anticancer activities, and participate in regulating immune response and blood lipid metabolism [23]. *Siraitia grosvenorii* residual extract inhibits Th2 and Th17 cytokines and enhances Th1 cytokines to repress allergic lung inflammation, and thus alleviates asthma [24]. *Siraitia grosvenorii* fruits extract exerts antihyperglycaemic effects by regulating the gut microbiota and its metabolites in type 2 diabetes mellitus rats [25]. This work uncovered that *Siraitia grosvenorii* had an antibacterial activity against *S*. *mutans*. The TCM compounds containing *Lonicera japonica*, *Chrysanthemum morifolium*, and *Siraitia grosvenorii* had slight influence on cell viability of primary human pharyngeal epithelial cells and inhibited the growth of *S*. *mutans*. TCM compound C3 reversed the inhibition effect of *S*. *mutans* on cell viability of primary human pharyngeal epithelial cells.

A previous study has reported that two active substances obtained from flowers of *Lonicera japonica*, 7-acetyl-8,9-dihydroxy thymol, and 7,8-dihydroxy-9-buyryl thymol play antibacterial effects on *Staphylococcus aureus*, *Escherichia coli*, *Micrococcus luteus*, and *Bacillus cereus* [26, 27]. *Chrysanthemum morifolium* extract represses the growth of different intestinal pathogenic bacteria, such as *Enterobacter*, *Enterococcus*, *Clostridium*, and *Bacteroides* [28]. The bioactive compounds aloe emodin, aloe-emodin acetate, and 5*α*,8*α*-epidioxy-24(R)-methylcholesta-6,22-dien-3*β*-ol and p-hydroxyl benzyl acid, extracted from *Siraitia grosvenorii* leaves limits the growth of oral bacterial species *S .mutans*, *Actinobacillus actinomycetemcomitans*, *Fusobacterium nucleatum*, and the yeast *Candida albicans* [29]. In general, there are many types of active substances in medicines, which active substance in C3 exerts the antibacterial effect still needs further research. In addition, *S. mutans* infection also causes other oral diseases, such as periodontitis and dental caries [30, 31]. *S. mutans* accounts for the largest proportion of oral flora and is one of the main components of dental plaque, which is the main pathogenic factor leading to periodontal disease [30]. *S. mutans* adheres, aggregates, produces acid, and destroys the hard tissue of the teeth in the form of plaque biofilm, eventually forming dental caries [32]. Thus, the TCM compound containing *Lonicera japonica*, *Chrysanthemum morifolium*, and *Siraitia grosvenorii* may have therapeutic effects on periodontitis and dental caries.

In summary, this work demonstrated that TCM compounds containing *Lonicera japonica*, *Chrysanthemum morifolium*, and *Siraitia grosvenorii* inhibited the growth of *S*. *mutans*. Thus, this study provides a theoretical basis for screening out the optimal compatibility of effective components in TCM mouthwash for relieving POST.

## Figures and Tables

**Table 1:** Uniform design experiment scheme of TCM compounds.

TCM compounds	*Lonicera japonica* (X); mg/mL	*Chrysanthemum morifolium* (Y); mg/mL	*Siraitia grosvenorii* (Z); mg/mL
C1	40	15	25
C2	45	25	20
C3	50	10	15
C4	55	20	10
C5	60	30	30

## Data Availability

All datasets used in this study are included in the manuscript.

## Supplementary Materials

Supplementary Materials Supplementary figure 1: the antibacterial activity of sodium citrate and Tween-80 on *S. mutans* was measured.
