# Supplementary figures and images for: Traditional Chinese Medicine Compounds Containing Lonicera japonica, Chrysanthemum morifolium, and Siraitia grosvenorii Inhibits the Growth of Streptococcus mutans
Source: Evid Based Complement Alternat Med. 2022 Oct 13;2022:5802343. doi: 10.1155/2022/5802343 (PMC9584668; doi:10.1155/2022/5802343)

Control

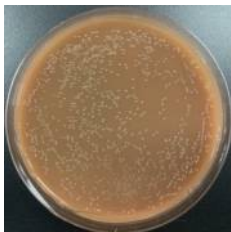

H<sub>2</sub>O

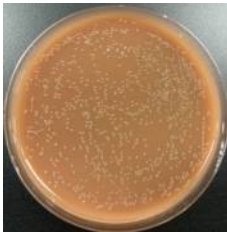

Sodium citrate  
+Tween-80

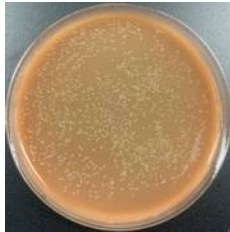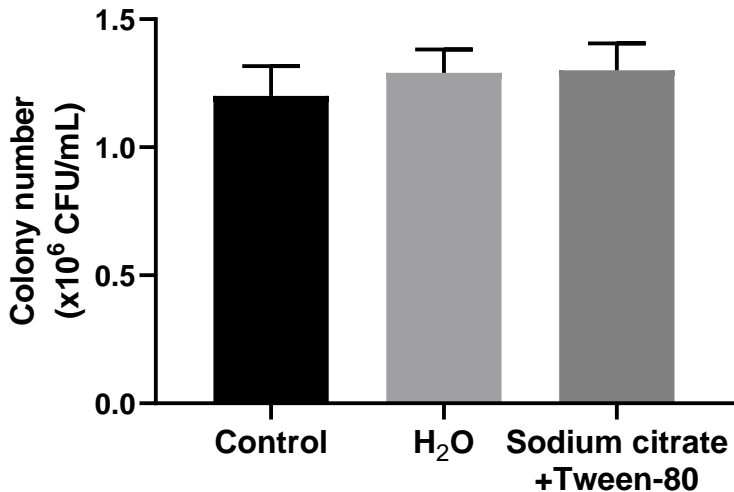

Supplement: Supplementary Materials — Supplementary figure 1: the antibacterial activity of sodium citrate and Tween-80 on S. mutans was measured. [file 5802343.f1.zip › Supplementary Figure 1.pdf]
